# Building a resilient coexistence with wildlife in a more crowded world

**DOI:** 10.1093/pnasnexus/pgad030

**Published:** 2023-02-01

**Authors:** Neil H Carter, John D C Linnell

**Affiliations:** University of Michigan, School for Environment and Sustainability, Ann Arbor, MI 48109, USA; Norwegian Institute for Nature Research, Lillehammer 2624, Norway; Department of Forestry and Wildlife Management, Inland Norway University of Applied Sciences, Evenstad 2480, Norway

## Abstract

There is an urgent need to sustainably coexist with wildlife. However, realizing this goal is hampered by scant understanding of the processes that facilitate and maintain coexistence. Here, we synthesize human–wildlife interactions into eight archetypal outcomes, from *eradication* to *sustained co-benefits*, which collectively serve as a heuristic for forms of coexistence across a wide range of species and systems worldwide. We utilize resilience theory to elucidate how and why human–wildlife systems shift between these archetypes, yielding insights on research and policy priorities. We underscore the importance of governance structures that actively enhance the resilience of coexistence.

## Introduction

A majority of the earth's surfaces—including urban, rural, wildland, terrestrial, and aquatic realms, and both protected and unprotected areas—are shared by humans and wildlife (1). These shared areas are no longer exceptions but represent common novel ecosystems (2), characterized by diverse and dynamic interactions between people and wildlife. Some of these interactions can involve people negatively impacting wildlife and wildlife negatively impacting human livelihoods (3). These negative interactions have motivated many previous studies around human–wildlife conflict (4). The increasing prevalence of these shared areas and their implications for biodiversity conservation and human well-being begs the question: how to respond to conflicts and achieve sustainable coexistence in these shared spaces?

The answer to this question is complex and will likely be determined by patterns of mutual adaptations between people and wildlife (5). Coadaptation in this context refers to humans and wildlife changing their behavior, learning from experience, and pursuing their own interests with respect to each other. On one hand, for example, herbivores can learn to feed on agricultural crops, or carnivores can learn to prey on domestic livestock, fueling human antagonism of those wild species (6). Humans have long responded to negative impacts from wildlife by lethally removing them, jeopardizing the long-term viability of many wild species worldwide (7). On the other hand, wildlife can provide people with ecosystem services, economic opportunities, and enriching relations with nature (8, 9). Human actions in turn promote wildlife persistence through environmental conservation policies, habitat restoration, reintroduction, and supplemental feeding (10, 11). The disparate outcomes of human–wildlife interactions—from species eradication to sustained co-benefits—motivate a discussion on how we can govern these interactions to lead to a sustainable system that balances costs and benefits in an elusive state that is increasingly being referred to as “coexistence” (5, 8).

**Fig. 1.**
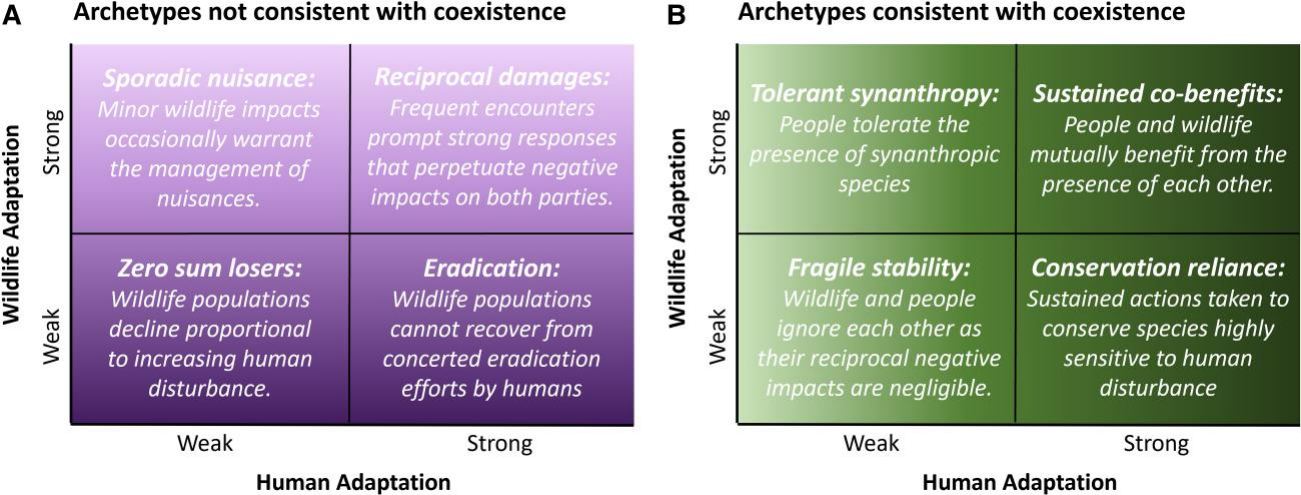
Archetypal outcomes of coadaptation between people and wildlife in shared areas. The outcomes are related to the degree to which people and wildlife adapt to each other. Outcomes in purple (A) are characterized largely by negative impacts and are not considered a state of coexistence (darker shades at bottom are farther from coexistence). In contrast, green outcomes (B) are characterized largely by positive impacts and, if sustained, can be referred to as states of coexistence (darker shades at right closer to coexistence). These archetypes are intended as a heuristic to understand human–wildlife systems and their positioning relative to the goal of achieving coexistence.

A widely accepted definition of coexistence is that it is a “dynamic but sustainable state in which humans and wildlife co-adapt to living in shared areas where human interactions with wildlife are governed by effective institutions that ensure long-term wildlife population persistence, social legitimacy, and tolerable levels of risk.” (5) We build on this conceptualization by synthesizing coadaptations between people and wildlife across a diverse array of conditions into a set of eight archetypal outcomes (Fig. 1), each of which we then position relative to the goal of achieving coexistence. We also establish links between these archetypes, and their dynamics, to system resilience—a powerful theoretical framework emerging in sustainability science. We conclude by highlighting priority research and policy directions for guiding the transformative change necessary to achieve human–wildlife coexistence.

## Archetypal outcomes of coadaptation

Systems of human–wildlife interactions are complex, shaped by past social and environmental conditions and their local contexts (12). Yet, they also share structural properties and processes of change, namely mutual adaptations between people and wildlife (13–15). Based on our combined experience of working on wildlife conservation issues, we synthesized these shared features of human–wildlife systems into eight archetypal outcomes, four of which we consider to not be consistent with coexistence (Fig. 1A) and four that are consistent with coexistence (Fig. 1B). The archetypes represent outcomes at the level of the whole system, focusing on wildlife populations and human societies. Below we describe the archetypes as belonging to four quadrants: (1) outcomes from weak coadaptations; (2) outcomes from strongly adapting wildlife; (3) outcomes from strongly adapting humans; and (4) outcomes from strong coadaptations (Fig. 1).

People and wildlife may weakly adapt to each other. As wildlife are unable to exploit human-modified areas in these cases, the populations of these animals can decline as their habitats are lost or degraded due to human development and encroachment. Thus, these species are *zero-sum losers* referring to situations in which whatever is gained by one side (i.e. humans) is lost by the other (i.e. wildlife). For example, many amphibian species represent this archetype, as their decline is strongly associated with human land uses, such as the expansion of agriculture at the expense of wetlands (16). For z*ero-sum losers*, humans have not constrained, modified, or adapted their behaviors to allow wildlife to persist or are even aware that human actions are directly leading to species decline. However, coadaptations can reach a *fragile stability* when human actions do not directly affect wildlife populations, and the presence of wildlife is tolerated, or even ignored, by people. For example, some bat species can persist in shared areas (17), though their capacity to behaviorally adapt to human disturbance may be limited, and their benefits and costs to human communities are typically overlooked. Although a more positive outcome than *zero sum losers*, this archetype is a fragile coexistence because it depends on human–wildlife interactions remaining relatively unchanged. This archetype is thus unlikely to persist in the face of rapid social and environmental change. For example, human tolerance to wildlife may be dependent on specific cosmologies and worldviews that are under threat from modernity (18).

Wildlife can also demonstrate strong adaptation to humans in shared areas. Some species can exhibit high behavioral plasticity, adjusting their behaviors to persist in human-modified areas. That many species are becoming more nocturnal or reduce their movements with increasing human disturbance is an example of such plasticity (19, 20), though the impacts of those shifts in daily activity patterns on those species can be positive or negative depending on the ecological context. Some species thrive in human-occupied areas, and their presence is likewise tolerated by people. Barn swallows illustrate this archetype of *tolerant synanthropy*, whereby they benefit from nesting sites and foraging opportunities associated with agricultural and urbanized areas, and people tolerate their presence (21). Occasionally, however, synanthropic wildlife cause nuisances, such as damage to property, or pose risks to domestic species through disease transmission. This archetype of *sporadic nuisance* includes weak adaptation by humans in the form of nuisance management (e.g. aversive conditioning, translocations, or limited culling); a response that is largely ignored by the broader public and unlikely to significantly reduce those populations of wildlife in the long term. Common raccoons (*Procyon lotor*) in North America are an example of this archetype. They are attracted to, and benefit from, human food sources and sometimes subjected to pest management due to impacts on human interests (e.g. zoonotic disease, property damage); though these management actions are ad hoc, localized, and rarely diminish the overall benefit of shared areas to raccoon populations (22). Although wildlife species can persist in human-occupied areas for a long time as a *sporadic nuisance*, in general, the risks from the wildlife species are not tolerated and outweigh the perceived benefits.

Humans may also demonstrate strong adaptation to wildlife. When viewed as significant competitors for space and food, or as detrimental to well-being, humans can resort to intentionally eradicating species from whole areas. Historically, large carnivores fell into the *eradication* archetype, with the public endorsing a variety of efforts—e.g. hunting, trapping, poisoning—to lethally remove these animals and pave the way for human settlement. Indeed, governments in the US and Europe historically paid bounties to facilitate large-scale eradication of large carnivores (23, 24). Some vulture species are more recent examples, whereby people in various regions indiscriminately poison these animals to remove them from the landscape, either because they are vilified or considered a threat to agricultural production (25). In contrast, people can mobilize conservation actions and policies to mitigate or reverse the negative impacts of past or ongoing human activities on wildlife. For wild species that are highly sensitive to human activities or are already threatened with extinction, sustained conservation actions are required to facilitate species persistence. Such outcomes fall within the *conservation reliance* archetype and are illustrated by tigers (*Panthera tigris*), a globally endangered species that requires strong conservation policies across their Asian range in order to persist alongside high human densities (26).

Strong coadaptation between wildlife and people can lead to divergent outcomes. By virtue of adapting to and exploiting human-modified areas, some wildlife species can cause significant economic losses or threaten human safety. People consequently adapt to these impacts by employing various lethal and nonlethal strategies to minimize these impacts. Such patterns of coadaptation can lead to the *reciprocal damages* archetype when techniques or policies to reduce negative impacts from wildlife are not adopted or largely ineffective, or human tolerance to risks posed by the species is low. Common leopards (*Panthera pardus*) in parts of South Asia are an example of this archetype. Leopards are considered highly adaptable to human-modified areas, persisting in places where other big cats have disappeared (27). This adaptability also means that they frequently encounter, and are often feared by, local communities. The consequent and intense persecution of leopards by humans is a widespread threat to this species (27). In contrast, coadaptations can lead to situations wherein wildlife and people mutually benefit each other at the level of the whole system. One example of the *sustained co-benefits* archetype is between deer species and people in North America and Europe. Deer benefit from our forestry (e.g. via abundant early successional habitat) and farming (e.g. increased plantings of winter crops), enabling their populations to thrive in those landscapes, while human communities benefit through hunting abundant deer species and wildlife viewing opportunities (28). Benefits from wildlife can be material and economic as well as spiritual and cultural (9, 29).

These eight archetypes range in how far or close they are to states of coexistence. The *zero-sum losers*, *eradication*, *sporadic nuisance*, and *reciprocal damages* archetypes cannot be regarded as states of coexistence (Fig. 1A), as human activities directly decrease the wildlife populations, and the sociopolitical context is either indifferent or actively hostile to the presence of the wildlife species in shared areas. In particular, the *zero-sum losers* and *eradication* archetypes are farthest from coexistence because the wildlife succumb to human pressures and, lacking any protection policies, are unable to persist in shared areas. In contrast, the *tolerant synanthropy*, *fragile stability*, *conservation reliance*, and *sustained co-benefits* archetypes are states consistent with most emerging conceptualizations of coexistence (Fig. 1B), as various sociopolitical and environmental forces interact to promote the presence of the wildlife species in the shared areas. In particular, the *conservation reliance* and *sustained co-benefits* archetypes are closest to coexistence, as they reflect social norms consisting of greater appreciation and stewardship of wildlife than those of the *tolerant synanthropy* and *fragile stability* archetypes (30). Social norms more favorable to wildlife underpin the institutional structures and policies that actively support the long-term conservation of wildlife in shared areas, despite the negative impacts to humans that may arise from doing so.

## Resilience of coadaptation archetypes

We posit that the archetypes have varying degrees of resilience—a capacity to sustain a shock and continue to function and cope with change (31). Mutual adaptations between people and wildlife can enhance the resilience of a certain archetype. For example, black bears (*Ursus americanus*) in urban environments often exploit garbage and other human food sources, despite being disturbed and harassed by people (32). These behaviors by bears increase during shortages of natural food resources, such as reduced production of various berries and nuts, implying anthropogenic foods are a source of subsidy when natural foods are scarce (33). These adaptations by bears may enable the resilience of the *sporadic nuisance* archetype when they pose intolerable risks for some people, prompting them to take action (e.g. hazing or lethal removal of problem animal). Likewise, mutual adaptations between people and spotted hyenas (*Crocuta crocuta*) in northern Ethiopia demonstrate the resilience of *tolerant synanthropy.* Hyenas in the region have been found to reach higher numbers in human-dominated areas compared to natural forest areas because they scavenge livestock carcasses left at urban waste dumps (34). Although hyenas can occasionally damage agricultural crops or kill livestock, they are tolerated in many places because they are viewed as vital sanitation agents and, in some cases, provide substantial tourist and cultural value (35, 36).

**Fig. 2.**
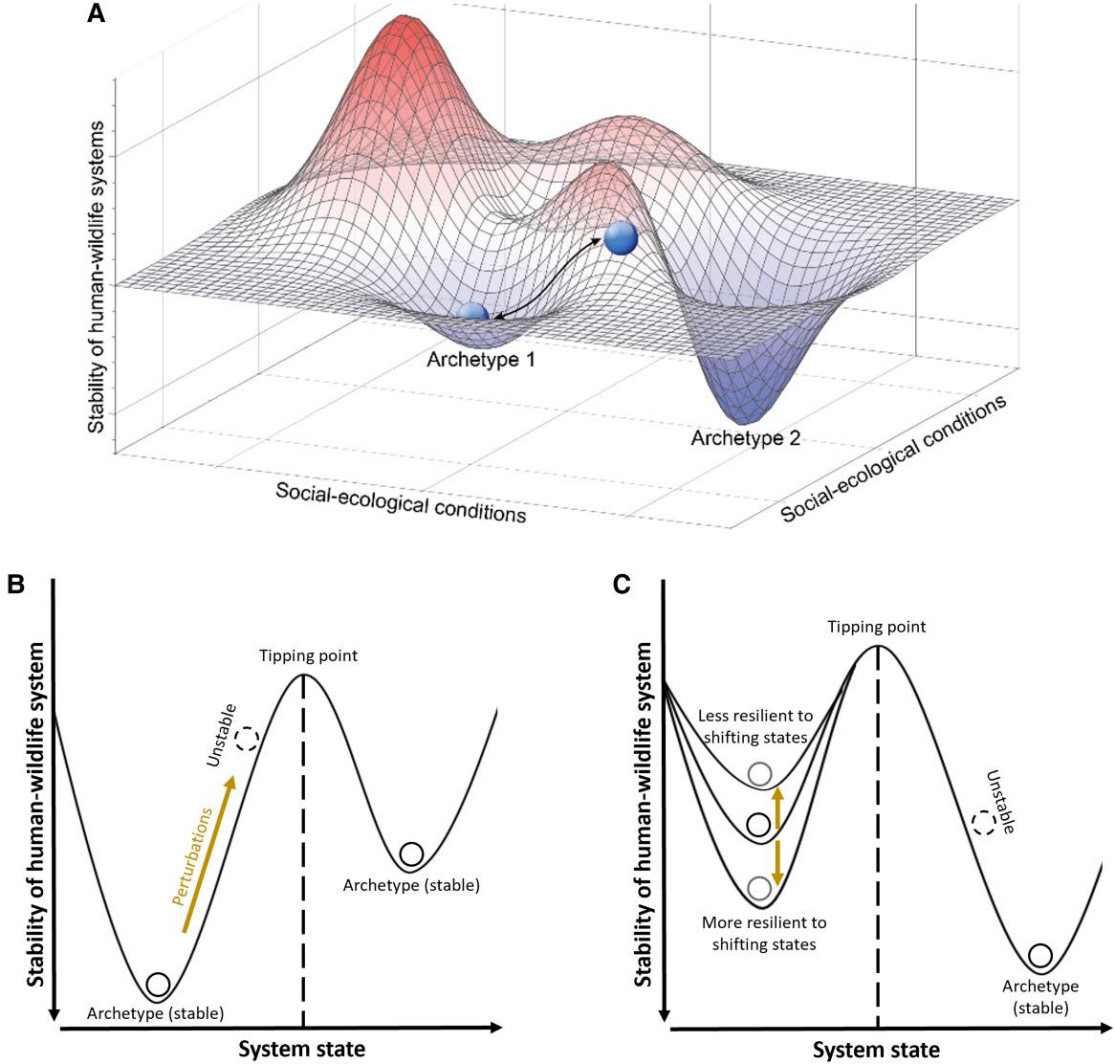
The hypothetical stability landscape for coexistence with hilltops and valleys. (A) Balls are found in the bottom of the valleys, which represent the coadaptation archetypes (i.e. stable states). These human–wildlife systems undergo perturbations in social-ecological conditions, which sometimes can shift them to different archetypes. (B) A 2-dimensional “ball and cup” representation of the stability landscape of two adjacent archetypes. Each archetype has a basin of attraction that describes its resilience to perturbations. Dashed circles indicate unstable systems. Large and persistent system perturbations, shown as brown arrows, can push systems over tipping points to other stable states (i.e. different archetypes). In addition to exogenous perturbations, (C) endogenous changes to the underlying sociopolitical and environmental contexts can cause a system to be more or less resilient to shifting to a different archetype.

Drawing from resilience theory (37), we consider these archetypes to comprise a “stability landscape”, which conceptualizes both the relative stability of different archetypes as well as how human–wildlife systems can move between different archetypes (Fig. 2A). Significant and cumulative perturbations can cause a shift from one archetype to another (Fig. 2B). Perturbations might include acute climate changes, such as drought, that alter the distribution of resources shared by both people and wildlife and cause increases in conflictual interactions. For example, a marine heat wave in 2015 led to changes in the migration timing of blue whales (*Balaenoptera musculus*) that caused them to die more often from collisions with ships off the coast of California, US (38), possibly shifting the system from *conservation reliance* toward *zero-sum losers*. Human actions can also significantly perturb systems of human–wildlife interactions. For example, the killing of Cecil the Lion in Zimbabwe by an American hunter ignited international furor over the practice of lion trophy hunting and may have hastened the adoption of significant new policies that restricted the importation of lion trophies by several countries (39). Those restrictions, in turn, likely have cascading consequences on human–lion interactions and land use; however, the exact nature of those impacts remain unclear (40). Furthermore, gradual changes to human values, demography, and socioeconomics can make archetypes more or less resilient to change (Fig. 2C), facilitating shifts to other archetypes. In the Zambezi region of Namibia, for example, the financial benefits that local communities and others are able to derive from conservation and sustainable use of African elephants may be shifting the system to *sustained co-benefits* rather than states characterized by negative impacts (41, 42), although *reciprocal damages* is common along edges of protected areas (43).

**Fig. 3.**
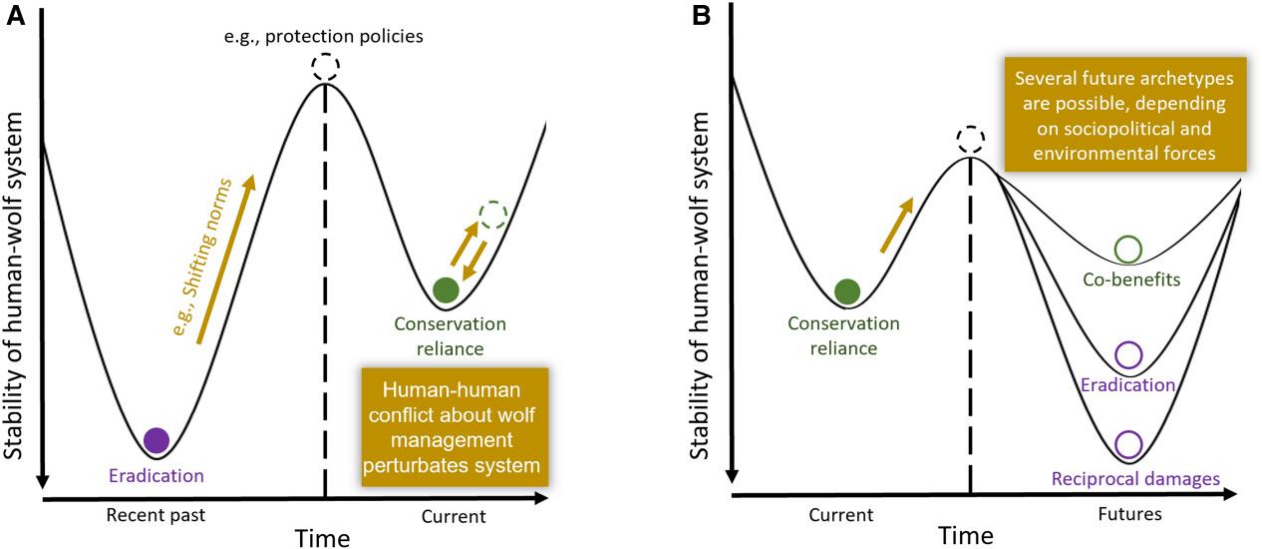
Hypothesized shifts through time of archetypes of the human–gray wolf system in the American West. Purple circles indicate archetypes moving away from coexistence, whereas green circles are those archetypes moving toward coexistence. (A) In the past, shifting norms around wildlife and more protective policies were “system perturbations” that pushed the system from eradication over a tipping point to the conservation reliance archetype. This current archetype experiences perturbations, for example, ongoing disagreement between different groups of people about the desired future of wolf populations in the region. (B) Perturbations in sociopolitical or environmental conditions can shift the current archetype to those that are farther or closer to a state of coexistence. Note that archetypes that are moving away from coexistence are more resilient to change than those moving toward coexistence, reflecting the notion that coexistence with wolves is not a self-organizing state but one requiring active and adaptable governance. For more information on this case, please refer to the Supplementary Materials.

Resilience theory also predicts that shifts between archetypes are characterized by tipping points or thresholds beyond which one archetype abruptly changes to another (Fig. 2B and C). These tipping points may relate to socioeconomic, political, or environmental factors. For example, crossing a tipping point of habitat loss or fragmentation can consistently increase human–wildlife conflicts to the point that they dominate system outcomes (44, 45). Of particular interest are social or political tipping points, as they can determine whether a system moves away from, or toward, coexistence (46, 47). For example, to manage a growing problem of crop losses by rhesus macaques (*Macaca mulatta*), the state government of Himachal Pradesh, India, permitted in 2010 the legal killing of macaques by afflicted farmers (48). The strong adaptive responses by both the macaques and humans, culminating in the political tipping point, shifted the system from *sporadic nuisance* toward *reciprocal damages*. Likewise, perceived threats to human interests in Idaho and Montana, US, by the growing gray wolf (*Canis lupus*) population has recently triggered severe policy responses designed to drastically reduce wolf numbers (49), possibly shifting the system from *conservation reliance* toward *reciprocal damages* or even *eradication—*the archetype farthest from coexistence (Fig. 3, Supplementary Materials). In contrast, the California condor (*Gymnogyps californianus*) is an example of a system moving toward coexistence. The condor was historically a *zero-sum loser* archetype, with a range of human activities—development, pesticide use, and hunting—causing rapid habitat loss and species decline. To reverse these losses, strong conservation policies were enacted and intensive human interventions, such as supplementing their populations by captive-reared individuals, have helped the condor evade extinction in the wild. These new policies, reflecting gradual changes in human worldviews and social norms, likely pushed the system over the tipping point from *zero-sum loser* to *conservation reliance.* Examples so far imply that a given human–wildlife system belongs to one archetype at a time; however, this is not always the case. Moose (*Alces alces*) in Scandinavia is a culturally important species that brings recreational and economic benefits to human communities but, at high densities, also leads to increased vehicle collisions and damages to commercial forestry (Supplementary Materials). Thus, human–moose systems appear to sit at the edge of *sustained co-benefits* and *reciprocal damages* depending on the costs and benefits flowing to different constituent groups.

Importantly, resilience is not synonymous with coexistence. Indeed, archetypes far from coexistence such as *eradication* can be highly resilient to change. For example, dingoes (*Canis dingo*) in Australia have historically been viewed as a pest species, subject to extermination programs across much of the continent (50). Although there is growing public discontent with lethal removal of dingoes and recognition of their potential positive ecological role, political structures maintain the status quo of dingo culling, still using inhumane methods in many regions (50, 51). For contentious species, such as large predators, archetypes consistent with coexistence may be less resilient than those not consistent with coexistence. We make this observation because removing animals that threaten human safety and livelihood has historically been the expedient solution to these risks. Whereas, building social norms of tolerance to these species, despite their risks, and instituting protective policies have involved opportunity costs (e.g. foregoing agriculture or livestock production in the species’ remaining habitats) and collective action (e.g. ballot initiatives, legislative bills). Thus, human interactions with some species may tend to “fall back” to a stable archetype not consistent with coexistence, such as *reciprocal damages*, without the governance structures and underlying tolerance necessary to sustainably promote the long-term persistence of these animals.

## Toward resilient coexistence

It is important to note that the resiliencies of the different archetypes are changing through time. For example, global economic growth and modernization have exerted tremendous pressures on natural systems, potentially pushing us into a biodiversity extinction crisis, but urbanization has also shifted our values toward wildlife (52). The goal of the conservation community should be to increase the resilience of those states consistent with coexistence and reduce the resilience of those that are not. The environmental sustainability literature underscores the central roles of adaptive capacity and transformation in changing system resilience in favor of desirable outcomes (53). We contend that adaptive capacity should extend to include coadaptations between people and wildlife. The capacity for wildlife to adapt to humans should be maintained (or enhanced) through human intervention, for example, by protecting wildlife priority habitats, dispersal corridors, and climate change refugia as well as limiting the harvest rate of species below “maximum yield” to buffer their populations against an uncertain future. Likewise, the capacity for humans to adapt to wildlife should be enhanced, for example, by retaining, relearning, and innovating new strategies for living with wildlife in a changing world. There are also numerous opportunities for governance institutions to facilitate mutual adaptations. For example, national or transnational institutions may set goals for wildlife conservation, while providing incentives to local communities to adopt and innovate strategies that meet those goals (54). Such incentive programs, if adequately designed, can reduce, or redistribute, the costs of coexistence for communities and build trust between communities and management authorities (55).

Achieving sustainable coexistence may require transformational change; that is, a fundamental, system-wide reorganization across technological, economic, and social factors (56, 57), making coexistence a norm (i.e. more resilient). Such change can be guided by applying priority interventions (levers) to key points of intervention (leverage points) (56, 58). Recent work suggests that key areas of leverage may involve reconnecting people to nature, restructuring institutions to provide more public engagement, and rethinking how knowledge is created and used in pursuit of coexistence (59). The connection people have to wildlife is important because it shapes the worldviews that underpin human action. However, sharing areas with species like large predators or elephants presents special cases, as they can pose real risks and costs to human well-being, including injury, fear, and psychological distress. We are also learning more about the positive, tangible impacts that these animals can have on human communities, such as saving lives and money through the reduction of vehicle–deer collisions or decreasing disease prevalence in livestock and people (36, 60), in addition to the various psychological benefits (61). The uneven distribution of the costs and benefits from risky wildlife drives human–human conflict over the desirability of coexisting with these animals. These ongoing human–human conflicts further underscore the need for transformative change in institutions. Because institutions guide and constrain human action, their restructuring can yield disproportionately large effects on the potential for achieving coexistence (62). Especially germane are those changes that ensure inclusive decision-making and the fair and equitable sharing of both costs and benefits from wildlife. One way to do this is to adopt a stronger emphasis on social justice by directly addressing inequities in conservation, including asymmetries in power and influence of actors in areas shared by humans and wildlife. Some also advocate for “multispecies” justice that jointly considers human and wildlife species (63). Finally, because human action builds on established knowledge, rethinking how we perceive and produce knowledge about wildlife could facilitate coexistence outcomes. This would entail, for example, developing coexistence strategies through the integration of different knowledge systems, including the sciences and indigenous and local knowledge, and the expansion of processes that engage with citizen science and the cogeneration of new knowledge (64, 65).

Leveraging resilience theory also opens new paths for future research on coexistence. One priority area is to identify the key set of variables that define resilience of the different archetypes. Given our definition of coexistence, these variables should at a minimum characterize three main components: wildlife population persistence (e.g. population size and demography, behavioral plasticity, and habitat features), institutional legitimacy (e.g. cost and benefit-sharing programs, representation in decision-making, and leadership), and tolerable levels of risk (e.g. wildlife services and disservices, human attitudes and norms about wildlife, and strategies for coping with risks) (66). Furthermore, understanding how different social and environmental factors perturb the system and drive shifts between archetypes can elucidate the dynamics of change in human–wildlife systems. For example, increasing societal tolerance to wildlife may more strongly drive coexistence outcomes than other factors if humans have a history of antipathy toward the species (6, 67). Although urbanization may enhance tolerance by reducing risks from wildlife, globalization may be eroding traditional tolerance values and practices toward some wildlife. Another important topic pertains to the nature of tipping points in human–wildlife systems. How common are they, and what social or environmental dimensions do they relate to? For example, economic tipping points may exist, such that people take action to remove wildlife (indicative of *reciprocal damages* archetype) if they can no longer afford to adapt to impacts from those animals, such as protecting livestock from predators. In contrast, increasing societal concern for the welfare of individual wildlife—i.e. deserving of care and compassion rather than only serving human interests (68)—may be indicative of an imminent social tipping point, beyond which activities that harm individual sentient animals are prohibited. Indeed, if there are early warning signs that a tipping point is approaching, that knowledge can be used to guide a system toward coexistence archetypes. Recent work has begun investigating the levers and leverage points for achieving coexistence with large predators (57). We recommend more work be conducted on this topic, generalizing across species and sites, as doing so can reveal pathways for transforming systems toward sustainable coexistence. Indeed, because coexistence archetypes may be associated with low resilience—requiring constant intervention—tackling the challenge of creating a sustainable funding and institutional framework for coexistence is a high priority.

## Concluding remarks

Coexisting with wildlife in shared areas is crucial for conserving biodiversity and maintaining (or improving) many dimensions of human well-being. The different archetypes of human–wildlife systems we present here provide a heuristic which researchers and practitioners can use to determine if a shared area is far or close to a state of coexistence. Lessons on strategies for coexisting with wildlife can be shared between human–wildlife systems in the same archetype to facilitate adaptive learning. Furthermore, shifts between archetypes (e.g. toward coexistence) are not random but likely reflect their resilience to social and environmental perturbations. Understanding the factors that influence their resilience promises to yield predictive insights on the efficacy of various conservation policies, such as protected area management and rewilding initiatives, as well as reveal early warning signs that coexistence is deteriorating. We recommend future research build on this synthesis in order to help us anticipate the future of human–wildlife interactions across diverse settings and under global changes.

## Supplementary Material

pgad030_Supplementary_Data

## Data Availability

No data are associated with this article.
