# Supplementary material for: Building a resilient coexistence with wildlife in a more crowded world
Source: PNAS Nexus. 2023 Feb 1;2(3):pgad030. doi: 10.1093/pnasnexus/pgad030 (PMC9991453; doi:10.1093/pnasnexus/pgad030)
Supplement: pgad030_Supplementary_Data [file pgad030_supplementary_data.docx]

**Supplementary Material**

Below are supplementary materials for the article “Building a Resilient Coexistence with Wildlife in a More Crowded World.” They include short descriptions of two cases—gray wolves in the American West and moose in Scandinavia—highlighting how co-adaptation archetypes dynamically change through time.

**Case 1: Human-Wolf Systems in American West**


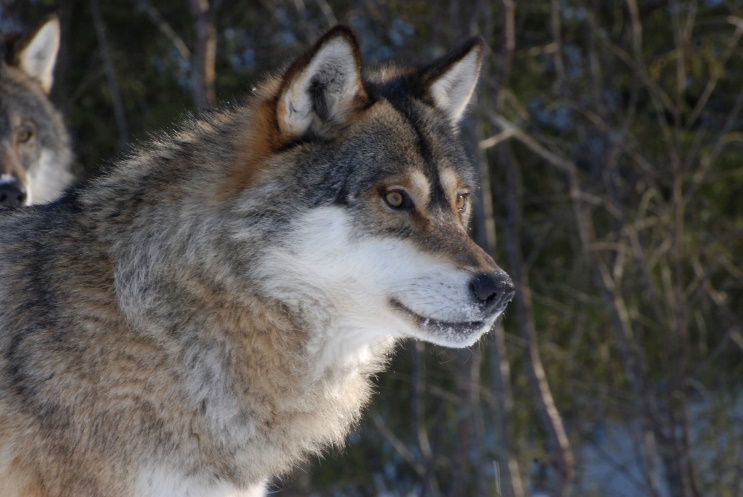
At the start of the 16^th^ century it has been estimated that there were 400,000 gray wolves (*Canis lupus*) living in what became the U.S.A (1). However, European colonists brought with them a fear and hatred of wolves based on cultural legacies from the Old World (2). Eventually it became the moral obligation of early pioneers to kill wolves, as the animal became symbolic of the threatening wilderness that settlers had to contend with. Wolves were fervently persecuted as European settlers expanded westward, with the efficiency of killing accelerated by the development of more effective firearms, poisons, and traps (3). The wolf decline was further driven by the decimation of native ungulate populations. By the late nineteenth and early twentieth centuries, government-sponsored eradication programs designed to protect livestock (and allow wild herbivores to recover) nearly eliminated wolves entirely from the contiguous USA (4). This period represents the *eradication* archetype (Fig. 3A in main text), characterized by a resilient system of co-adaptations between people and wolves that exacerbated wolf decline. In the second half of the 20^th^ century, documented environmental degradation and catastrophes mobilized public concern in the USA about human impacts on nature. Concomitant with this rising environmental movement was an increasingly favorable attitude toward wolves among the public, facilitated by more detailed field studies and popular stories about wolves. Urbanisation, shifting social norms along with the involvement of mainstream public conservation organizations helped lead to the listing of wolves in 1974 as “Endangered” under the U.S. Endangered Species Act of 1973. This and other conservation policies facilitated the reintroduction, population growth, and natural expansion of wolves in recent years. These policies pushed the system beyond a tipping point (at which they were no longer in short-term threat of extinction) and into the *conservation reliance* archetype (Fig. 3A in main text), characterized by wolf persistence contingent on federal-level conservation policies, constant management intervention and conflict management, and public support for their recovery. However, subsequent increases in livestock depredations by wolves, especially in the American West, and human killing of wolves has triggered contentious debates about how to manage the species in the future. The polarizing discourse around wolves reflects, in part, the diverging worldviews of a more diverse public, including those who largely prioritize livestock production or game species hunting and those that prioritize recovery of apex carnivores (5). It also reflects tensions over how decisions about wolves are made, e.g., centrally or locally. These human-human conflicts over wolves are system perturbations, which constantly test the resilience of the *conservation reliance* archetype. The system of human-wolf interactions in the Rocky Mountains, USA, has different possible futures (Fig. 3B in main text). The *sustained co-benefits* archetype, e.g., natural habitats are managed for wolves to increase wildlife tourism, could expand from places like Yellowstone National Park to other regions (6). Recent events may be early warning signs that the human-wolf system is moving toward (or has already passed) tipping points to other archetypes. For example, wolves in the US states Idaho and Montana had reached their recovery target and were delisted from the federal Endangered Species Act in 2011. This decision gave individual states the authority to manage wolves, and they promptly enacted laws to reduce wolf numbers. In 2021, Idaho and Montana passed laws that removed many restrictions on where, when, and how many wolves can be hunted, targeting up to 80-90% of the current wolf populations in those states. These laws permit the use of previously outlawed methods of killing wolves—e.g., snaring, baiting, and night hunting—and allow private contractors to implement these policies (7). This swing back to a system that has many of the hallmarks of the *eradication* archetype reflects its resilience. Whereas, in Colorado, the public narrowly voted in 2020 in favor of a ballot initiative to reintroduce wolves to the state (8), paving the way for a *conservation reliance* archetype in the area. Meanwhile, where wolves are rebounding in Oregon and Washington, increasing rates of human-wolf conflict suggest that a shift could occur between *conservation reliance* to *reciprocal damages*.

**Case 1 (wolf) references:**

1. J.-L. Martin, S. Chamaillé-Jammes, D. M. Waller, Deer, wolves, and people: costs, benefits and challenges of living together. *Biol. Rev. Camb. Philos. Soc.* **95**, 782–801 (2020).

2. S. H. Fritts, R. O. Stephenson, R. D. Hayes, L. Boitani, “Wolves and Humans” in *Wolves: Behavior, Ecology, and Conservation*, L. David Mech, L. Boitani, Eds. (University of Chicago Press, 2003), pp. 289–316.

3. L. D. Mech, The challenge and opportunity of recovering wolf populations. *Conserv. Biol.* **9**, 270–278 (1995).

4. B. J. Bergstrom, *et al.*, License to Kill: Reforming Federal Wildlife Control to Restore Biodiversity and Ecosystem Function. *Conservation Letters* **7**, 131–142 (2014).

5. N. H. Carter, *et al.*, Towards Human–Wildlife Coexistence through the Integration of Human and Natural Systems The Case of Grey Wolves in the Rocky Mountains, USA. *Human-Wildlife Interactions: Turning Conflict into Coexistence*, 384 (2019).

6. RRC Associates, “Greater Yellowstone wildlife related activity valuation study” (Institute for Tourism and Recreation Research, University of Montana, 2022).

7. E. A. Fitzgerald, Premature Gray Wolf Delisting. *Nat. Resour. J.* **62**, 183 (2022).

8. R. Niemiec, *et al.*, Rapid changes in public perception toward a conservation initiative. *Conservat Sci and Prac* **4** (2022).

**Case 2: Moose Management in Scandinavia**


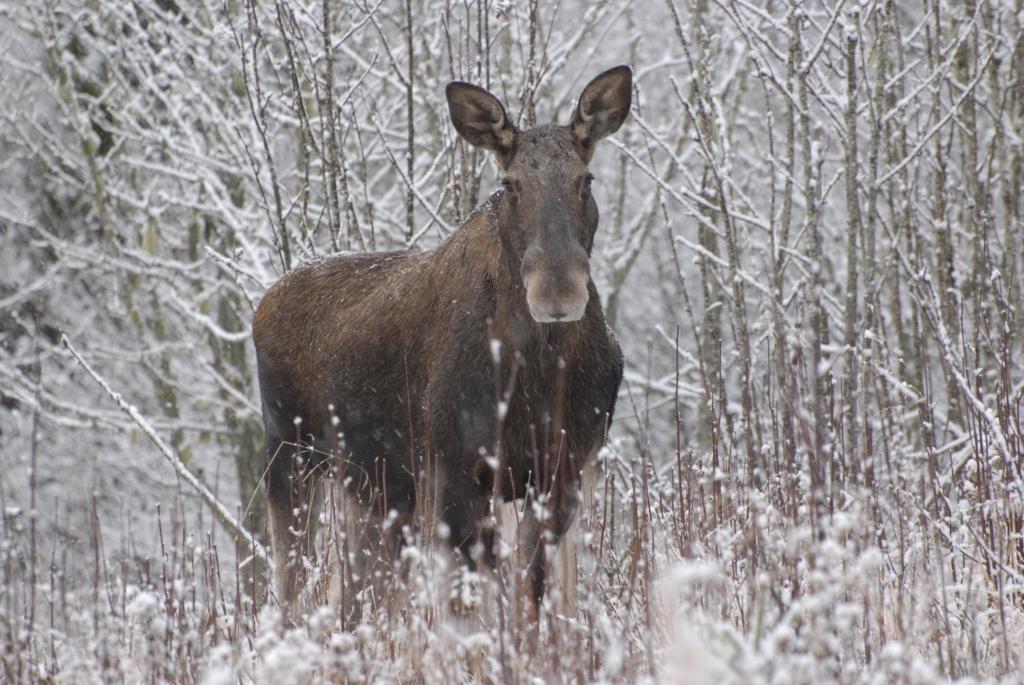
Moose (*Alces alces*) have been part of the Scandinavian fauna throughout the Holocene (1) and have long been important species for both human nutrition and spirituality, as indicated by archaeological remains such as the abundance of pitfall trap systems for hunting that lasted from the Mesolithic up until the Middle Ages and their presence in rock carvings that date from the Stone Age to the Bronze Age (2, 3). However, by the late 19^th^ century their populations had been dramatically reduced across Scandinavia by poorly regulated hunting pressure. Changes to hunting regulations and the near eradication of large carnivores permitted a slow increase in the early 20^th^ century, which dramatically increased post 1950 due to carefully planned hunting management and the increase forage availability after the introduction of clear-cutting logging practices. By the late 1980’s and early 1990’s populations had expanded to most of the Scandinavian peninsula and reached record high densities, with annual harvests of around 40,000 in Norway and 100,000 in Sweden (4–6). Moose therefore provide hunting opportunities for tens of thousands of recreational hunters and economic income for landowners through the sale of hunting licenses. Moose also constitute the major prey item for Scandinavia’s recovering wolf population (7). However, moose are also a source of human-wildlife conflict, with the most important being associated with vehicle collisions and damage to commercial forests and agricultural crops. Annually around 1,500 and 4,000 moose are killed in traffic collisions each year in Norway and Sweden, respectively, also causing massive vehicle damage and human injury and death as well as requiring expensive investment in highway fencing and other collision reducing measures. Estimating the economic cost of damage to forests and crops is challenging, but moose browsing is so extensive in some areas that it forces foresters to totally avoid some tree species and is viewed as being a major constraint and cost (4, 6).

Moose are currently a ubiquitous part of the Scandinavian multi-use landscape, occurring throughout almost all forested areas, including mixed agricultural-forest mosaics and the peri-urban-forest interface (8). In many ways the presence of the largest extant cervid species across the entire landscape is a classic example of coexistence. Moose remain a positive culturally significant species to this day. Moose are clearly able to adapt to human-modified landscapes and humans have clearly adapted to the presence of moose. There are currently no principled controversies about the actual presence of moose, however, there are often very heated local discussions about the appropriate densities of moose as different interest groups try to balance the diverse costs and benefits associated with the species (9, 10). Moose management in Scandinavia therefore sits on the border between *Sustained co-benefits* (moose benefit from forestry practices and agricultural crops, humans benefit from moose hunting and the aesthetic value of moose) and *Reciprocal damages* (moose populations are intensively managed through lethal control and humans experience damage to vehicles and personal injury as well as economic loss in forestry and agriculture) archetypes. The moose situation is rather typical of the management system for most wild herbivores across Europe, which currently are at higher levels than they have been for centuries, but where they are caught up in complex webs of interactions with humans that can represent costs and benefits depending on interests and perspectives (11). It is hard to see any future where the co-benefits could be received without the damages, implying that coexistence with moose will always remain a dynamic state at the borders between archetypes and where the science and art of wildlife management, and well-regulated hunting, is essential to walk the line between pest and resource.

**Case 2 (moose) references:**

1. J. Rosvold, R. Andersen, J. D. C. Linnell, A. K. Hufthammer, Cervids in a dynamic northern landscape: Holocene changes in the relative abundance of moose and red deer at the limits of their distributions. *Holocene* **23**, 1143–1150 (2013).

2. A. Hennius, Towards a refined chronology of prehistoric pitfall hunting in Sweden. *Eur. J. Archaeol.* **23**, 530–546 (2020).

3. K. Sognes, “Symbols in a changing world: rock-art and the transition from hunting to farming in mid-Norway” in *The Archaeology of Rock Art*, C. Chippindale, P. S. C. Tacon, Eds. (Cambridge University Press, 1998), pp. 146–163.

4. R. Andersen, E. Lund, E. J. Solberg, Saether B- E, “Ungulates and their management in Norway” in *European Ungulates and Their Management in the 21st Century*, M. Apollonio, R. Andersen, R. Putman, Eds. (Cambridge University Press, 2010), pp. 14–36.

5. S. Lavsund, T. Nygren, E. J. Solberg, Status of moose populations and challenges to moose management in Fennoscandia. *Alces* **39**, 109–130 (2003).

6. O. Liberg, R. Bergström, J. Kindberg, H. von Essen, “Ungulates and their management in Sweden” in *European Ungulates and Their Management in the 21st Century*, M. Apollonio, R. Andersen, R. Putman, Eds. (Cambridge University Press, 2010).

7. H. Sand, A. Eklund, B. Zimmermann, C. Wikenros, P. Wabakken, Prey selection of Scandinavian wolves: Single large or several small? *PLoS One* **11**, e0168062 (2016).

8. B. Cretois, *et al.*, Coexistence of large mammals and humans is possible in Europe’s anthropogenic landscapes. *iScience* **24**, 103083 (2021).

9. J. O. Olaussen, A. Skonhoft, A cost-benefit analysis of moose harvesting in Scandinavia. A stage structured modelling approach. *Res. Energy Econ.* **33**, 589–611 (2011).

10. T. Storaas, H. Gundersen, H. Henriksen, H. P. Andreassen, The economic value of moose in Norway – a review. *Alces* **37**, 97–107 (2001).

11. J. D. C. Linnell, *et al.*, The challenges and opportunities of coexisting with wild ungulates in the human-dominated landscapes of Europe’s Anthropocene. *Biol. Conserv.* **244**, 108500 (2020).
